# 2-[(Naphthalen-1-yl­methyl­idene)amino]-5-methyl­phenol

**DOI:** 10.1107/S1600536811034556

**Published:** 2011-08-27

**Authors:** Gabriella Orona, Vanessa Molinar, Frank R. Fronczek, Ralph Isovitsch

**Affiliations:** a Department of Oral Biology and Medicine, University of California, Los Angeles, 10833 Le Conte Avenue, Los Angeles, CA 90095-1668, USA; b Texas Tech University Health Science Center, Paul L. Foster School of Medicine, 5001 El Paso Drive, El Paso, TX 79905, USA; c Department of Chemistry, Louisiana State University, Baton Rouge, LA 70803, USA; d Department of Chemistry, Whittier College, 13406 Philadelphia Street, Whittier, CA 90601, USA

## Abstract

The title compound, C_18_H_15_NO, is a Schiff base prepared from an acid-catalyzed condensation reaction between 1-naphthaldehyde and 6-amino-*m*-cresol. Intra­molecular hydrogen bonding occurs *via* an O—H⋯N inter­action, generating an *S*(5) ring motif. Neighboring phenol groups participate in inter­molecular hydrogen bonding through an O—H⋯O inter­action, forming chains. The O atom of the phenol group also participates in an intermolecular C—H⋯O interaction with an H atom of one of the naphthalene rings. The C—N=C—C torsion angle between the phenol and naphthalene rings is −179.8 (2)°. Crystal packing involves stacks with the mol­ecules inter­acting through the π-systems of the C=N with both the phenol system and one of the naphthalene rings.

## Related literature

For related structures, see: De *et al.* (2008 ▶); Villalpando *et al.* (2010 ▶); Yildz *et al.* (2005 ▶). For bond-length data, see Allen *et al.* (1987 ▶). For background to the synthesis of Schiff bases, see: Borisova *et al.* (2007 ▶). For background to the use of Schiff bases in solar energy collection, see: Mak *et al.* (2009 ▶). For background to the inter­molecular inter­actions of π-systems, see: Jennings *et al.* (2006 ▶); Zhang *et al.* (2006 ▶). For a description of hydrogen-bonding motifs, see: Bernstein *et al.* (1995 ▶).
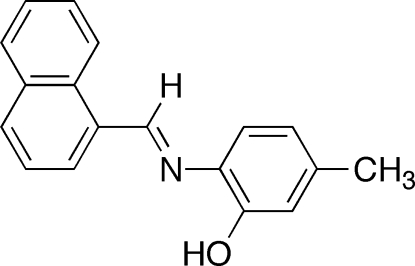


## Experimental

### 

#### Crystal data

C_18_H_15_NO
*M* *_r_* = 261.31
Orthorhombic, 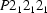

*a* = 4.8246 (10) Å
*b* = 9.766 (2) Å
*c* = 28.024 (7) Å
*V* = 1320.4 (5) Å^3^
*Z* = 4
Mo *K*α radiation
μ = 0.08 mm^−1^
*T* = 90 K
0.25 × 0.17 × 0.10 mm

#### Data collection

Nonius KappaCCD (with an Oxford Cryosystems Cryostream cooler) diffractometer
14676 measured reflections
1552 independent reflections
1169 reflections with *I* > 2σ(*I*)
*R*_int_ = 0.034

#### Refinement

*R*[*F* ^2^ > 2σ(*F* ^2^)] = 0.041
*wR*(*F* ^2^) = 0.090
*S* = 1.05
1552 reflections
185 parameters
H atoms treated by a mixture of independent and constrained refinement
Δρ_max_ = 0.19 e Å^−3^
Δρ_min_ = −0.17 e Å^−3^

### 

Data collection: *COLLECT* (Nonius 2000 ▶); cell refinement: *DENZO* and *SCALEPACK* (Otwinowski & Minor, 1997 ▶); data reduction: *DENZO* and *SCALEPACK*; program(s) used to solve structure: *SIR97* (Altomare *et al.*, 1999 ▶); program(s) used to refine structure: *SHELXL97* (Sheldrick, 2008 ▶); molecular graphics: *ORTEP-3 for Windows* (Farrugia, 1997 ▶); software used to prepare material for publication: *SHELXL97*.

## Supplementary Material

Crystal structure: contains datablock(s) I, global. DOI: 10.1107/S1600536811034556/lr2024sup1.cif

Structure factors: contains datablock(s) I. DOI: 10.1107/S1600536811034556/lr2024Isup2.hkl

Supplementary material file. DOI: 10.1107/S1600536811034556/lr2024Isup3.cml

Additional supplementary materials:  crystallographic information; 3D view; checkCIF report

## Figures and Tables

**Table 1:** Hydrogen-bond geometry (Å, °)

*D*—H⋯*A*	*D*—H	H⋯*A*	*D*⋯*A*	*D*—H⋯*A*
O1—H1*O*⋯O1^i^	0.87 (3)	2.17 (3)	2.916 (2)	143 (2)
O1—H1*O*⋯N1	0.87 (3)	2.24 (3)	2.701 (3)	113 (2)
C2—H2⋯O1^i^	0.95	2.53	3.382 (3)	150
C18—H18*C*⋯*Cg*^ii^	0.98	2.57	3.504 (3)	160

Symmetry codes: (i) 
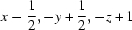
; (ii) 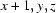.

**Fig. 1.**
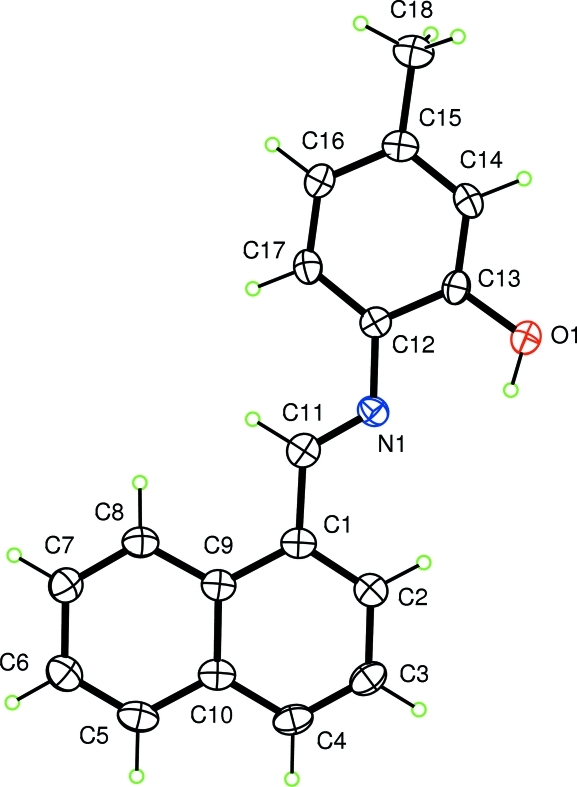
ORTEP plot of the title compound with displacement ellipsoids at the 50% probability level. H atoms are shown with arbitrary radius.

## supplementary crystallographic information

### Comment

Schiff bases are traditionally prepared *via* an acid-catalyzed condensation reaction between an aniline derivative and a ketone or aldehyde (Borisova *et al.*, 2007). Our research examines the photophysics of polycyclic aromatic hydrocarbon Schiff bases and their metal complexes toward the goal of applying them to solar energy collection (Mak *et al.*, 2009).

The structure of the title compound is shown in Figure 1. The atoms of the central double bond (N1—C11) have a bond length and bond angles that indicate their *sp*^2^ hybrid character (Allen *et al.*, 1987). For example, N1—C11 has a length of 1.273 (3) Å. Angles about the N1—C11 double bond, C11—N1—C12 and N1—C11—C1, are 121.1 (2) and 122.5 (2)° respectively. The molecule deviates sligthly from planarity with C12—N1—C11—C1 torsion angle of -179.8 (2)° Other observed bond angles and lengths correspond well with those of similar phenolic Schiff bases. (De *et al.* , 2008; Villalpando *et al.*, 2010).

Intramolecular hydrogen bonding was observed in the title compound in an O—H···N (O1H—N1) interaction with a H···N distance of approximately 2.2 Å and a O···N distance of 2.701 (2) Å, generating S(5) ring motifs. Neighboring OH (O1H) groups participated in intermolecular hydrogen bonding through an O—H···O (at *x* - 1/2, 1/2 - *y*, 1 - *z*) interaction with an H···O distance of approximately 2.2 Å and an O···O distance of 2.916 (2) Å, with an O—H···O bond angle of 143 (2)°. This type of intra- and intermolecular hydrogen bonding has been observed in other Schiff bases (Yildz *et al.*, 2005; Villalpando *et al.*, 2010).

Crystals of the title compound were composed of stacks of the molecule engaged in various intermolecular π-interactions. A π-π interaction occurred through the central double bond (N1—C11) and one of the naphthalene rings (C1—C2—C3—C4—C10—C9) with a N···*Cg* (at *x* + 1, *y*, *z*) distance of 3.336 (2) Å. Another π-π interaction occurred through the central double bond (N1—C11) and the phenol ring (C12—C17) with a N···*Cg* (at *x* - 1, *y*, *z*) distance of 3.491 (2) Å. A C—H···π interaction was observed between the methyl hydrogen atoms (C18—H) and the phenol ring (C12—C17) with a H···*Cg* (at *x* + 1, *y*, *z*) distance of approximately 2.6 Å and a C18···*Cg* distance of 3.504 (2) Å, with a C18—H···*Cg* angle of 160°.

Stacks of the molecule interacted in two ways. The first being the intermolecular O—H···O hydrogen bonding described above. The second being a C—H···O (C2H—O1) interaction with a H···O (at *x* - 1/2, 1/2 - *y*, 1 - *z*) distance of approximately 2.5 Å and a C···O distance of 3.382 (3) Å with a C—H···O bond angle of 150°. These types of interactions have been seen in other aromatic systems (Jennings *et al.*, 2006; Zhang *et al.*, 2006).

### Experimental

Synthetic procedures were carried out using standard techniques. Solvents and reagents were purchased from Sigma-Aldrich or Acros Organics and used as received. The melting point was determined in an open capillary and is uncorrected. The ^1^H-NMR spectrum was recorded on a Jeol ECX 300 MHz s pectrometer using TMS as the internal standard. The IR spectrum was recorded as a KBr disk on a JASCO 460 F T—IR.

In a 25 ml roundbottom flask, 1-napthaldehyde (8.7 ml, 0.64 mmol) was added to 6-amino-*m*-cresol (0.087 g, 0.70 mmol) along with four drops of concentrated acetic acid and 10 ml me thanol. The solution was refluxed for 2 h. When the reaction time was complete, the reaction volume was reduced by half and allowed to cool slowly. The resultant dark orange crystals were vacuum filtered and air dried. 0.102 g of product were obtained, which is a 61% yield.

MP: 111–113°C. IR (KBr): 3053, 1696, 1510 cm^-1. 1^H NMR (300 MHz, CDCl_3_) 9.38 (s, 1 H), 8.85 (d, 1 H), 8.17 (d, 1 H), 7.97 (m, 2 H), 7.61 (m, 3 H), 7.29 (m, 2 H), 6.89 (d, 1 H), 6.77 (m, 1 H), 2.37 (s, 3 H) p.p.m.. EI—GC—MS: m/*z*= 261.1. TLC (silica, CH_2_Cl_2_): *R_f_*= 0.77.

### Refinement

All H atoms on C were placed in calculated positions, guided by difference maps, with C—H bond distances 0.95–0.98 Å, and thereafter refined as riding. A torsional parameter was refined for the Me group. The hydroxy H atom coordinates were refined. Isotropic displacement parameters for H were assigned as *U*_iso_=1.2*U*_eq_, (1.5 for methyl and OH). Friedel pairs were averaged.

### Crystal data

**Table:**

C_18_H_15_NO	*F*(000) = 552
*M**_r_* = 261.31	*D*_x_ = 1.314 Mg m^−^^3^
Orthorhombic, *P*2_1_2_1_2_1_	Mo *K*α radiation, λ = 0.71073 Å
Hall symbol: P 2ac 2ab	Cell parameters from 1569 reflections
*a* = 4.8246 (10) Å	θ = 2.5–26.0°
*b* = 9.766 (2) Å	µ = 0.08 mm^−^^1^
*c* = 28.024 (7) Å	*T* = 90 K
*V* = 1320.4 (5) Å^3^	Fragment, orange
*Z* = 4	0.25 × 0.17 × 0.10 mm

### Data collection

**Table:**

Nonius KappaCCD (with an Oxford Cryosystems Cryostream cooler) diffractometer	1169 reflections with *I* > 2σ(*I*)
Radiation source: fine-focus sealed tube	*R*_int_ = 0.034
graphite	θ_max_ = 26.1°, θ_min_ = 2.9°
ω and φ scans	*h* = −5→5
14676 measured reflections	*k* = −11→12
1552 independent reflections	*l* = −34→34

### Refinement

**Table:**

Refinement on *F*^2^	Primary atom site location: structure-invariant direct methods
Least-squares matrix: full	Secondary atom site location: difference Fourier map
*R*[*F*^2^ > 2σ(*F*^2^)] = 0.041	Hydrogen site location: inferred from neighbouring sites
*wR*(*F*^2^) = 0.090	H atoms treated by a mixture of independent and constrained refinement
*S* = 1.05	*w* = 1/[σ^2^(*F*_o_^2^) + (0.0412*P*)^2^ + 0.1692*P*] where *P* = (*F*_o_^2^ + 2*F*_c_^2^)/3
1552 reflections	(Δ/σ)_max_ < 0.001
185 parameters	Δρ_max_ = 0.19 e Å^−^^3^
0 restraints	Δρ_min_ = −0.17 e Å^−^^3^

### Special details

**Table:**

Geometry. All e.s.d.'s (except the e.s.d. in the dihedral angle between two l.s. planes) are estimated using the full covariance matrix. The cell e.s.d.'s are taken into account individually in the estimation of e.s.d.'s in distances, angles and torsion angles; correlations between e.s.d.'s in cell parameters are only used when they are defined by crystal symmetry. An approximate (isotropic) treatment of cell e.s.d.'s is used for estimating e.s.d.'s involving l.s. planes.
Refinement. Refinement of *F*^2^ against ALL reflections. The weighted *R*-factor *wR* and goodness of fit *S* are based on *F*^2^, conventional *R*-factors *R* are based on *F*, with *F* set to zero for negative *F*^2^. The threshold expression of *F*^2^ > σ(*F*^2^) is used only for calculating *R*-factors(gt) *etc*. and is not relevant to the choice of reflections for refinement. *R*-factors based on *F*^2^ are statistically about twice as large as those based on *F*, and *R*- factors based on ALL data will be even larger.

### Fractional atomic coordinates and isotropic or equivalent isotropic displacement parameters (Å^2^)

**Table:**

	*x*	*y*	*z*	*U*_iso_*/*U*_eq_	
O1	0.7221 (4)	0.32980 (18)	0.49100 (6)	0.0319 (5)	
H1O	0.572 (6)	0.287 (3)	0.4823 (10)	0.048*	
N1	0.4847 (4)	0.3060 (2)	0.40411 (7)	0.0233 (5)	
C1	0.1310 (5)	0.1903 (2)	0.35845 (9)	0.0242 (6)	
C2	0.0777 (5)	0.0959 (2)	0.39380 (9)	0.0268 (6)	
H2	0.1808	0.0994	0.4226	0.032*	
C3	−0.1273 (6)	−0.0059 (2)	0.38802 (10)	0.0292 (7)	
H3	−0.1612	−0.0694	0.4130	0.035*	
C4	−0.2771 (6)	−0.0136 (3)	0.34679 (10)	0.0293 (7)	
H4	−0.4124	−0.0835	0.3431	0.035*	
C5	−0.3912 (6)	0.0766 (3)	0.26682 (9)	0.0300 (7)	
H5	−0.5287	0.0078	0.2631	0.036*	
C6	−0.3497 (6)	0.1687 (3)	0.23107 (9)	0.0308 (6)	
H6	−0.4587	0.1644	0.2029	0.037*	
C7	−0.1459 (6)	0.2700 (2)	0.23600 (9)	0.0291 (7)	
H7	−0.1150	0.3330	0.2107	0.035*	
C8	0.0095 (5)	0.2794 (2)	0.27681 (9)	0.0269 (6)	
H8	0.1435	0.3502	0.2797	0.032*	
C9	−0.0265 (5)	0.1851 (2)	0.31488 (9)	0.0237 (6)	
C10	−0.2324 (5)	0.0814 (2)	0.30962 (9)	0.0241 (6)	
C11	0.3451 (6)	0.2966 (3)	0.36571 (9)	0.0270 (6)	
H11	0.3798	0.3602	0.3408	0.032*	
C12	0.6877 (5)	0.4091 (2)	0.40997 (8)	0.0209 (6)	
C13	0.8054 (5)	0.4165 (2)	0.45550 (9)	0.0229 (6)	
C14	1.0077 (5)	0.5127 (2)	0.46633 (9)	0.0250 (6)	
H14	1.0852	0.5149	0.4975	0.030*	
C15	1.0982 (5)	0.6057 (2)	0.43233 (9)	0.0242 (6)	
C16	0.9835 (5)	0.5979 (3)	0.38650 (9)	0.0254 (6)	
H16	1.0429	0.6604	0.3626	0.030*	
C17	0.7852 (5)	0.5008 (2)	0.37545 (9)	0.0256 (6)	
H17	0.7138	0.4963	0.3439	0.031*	
C18	1.3138 (5)	0.7117 (2)	0.44451 (9)	0.0314 (7)	
H18A	1.2784	0.7477	0.4766	0.047*	
H18B	1.3051	0.7867	0.4213	0.047*	
H18C	1.4983	0.6698	0.4436	0.047*	

### Atomic displacement parameters (Å^2^)

**Table:**

	*U*^11^	*U*^22^	*U*^33^	*U*^12^	*U*^13^	*U*^23^
O1	0.0329 (11)	0.0365 (11)	0.0263 (10)	−0.0067 (10)	−0.0023 (9)	0.0079 (9)
N1	0.0195 (11)	0.0259 (11)	0.0246 (12)	0.0010 (11)	−0.0005 (10)	−0.0013 (10)
C1	0.0213 (14)	0.0214 (13)	0.0300 (14)	0.0024 (13)	0.0029 (13)	−0.0045 (12)
C2	0.0269 (15)	0.0248 (13)	0.0288 (15)	0.0030 (13)	−0.0005 (12)	−0.0026 (12)
C3	0.0310 (16)	0.0219 (13)	0.0348 (17)	0.0017 (14)	0.0077 (15)	0.0024 (13)
C4	0.0268 (16)	0.0214 (13)	0.0397 (17)	−0.0012 (13)	0.0065 (14)	−0.0023 (13)
C5	0.0240 (15)	0.0280 (13)	0.0379 (17)	−0.0013 (13)	0.0028 (13)	−0.0108 (13)
C6	0.0310 (16)	0.0328 (14)	0.0286 (15)	0.0021 (15)	0.0005 (14)	−0.0073 (13)
C7	0.0311 (16)	0.0298 (14)	0.0263 (16)	0.0010 (14)	0.0040 (13)	−0.0025 (12)
C8	0.0253 (15)	0.0243 (13)	0.0310 (15)	−0.0009 (12)	0.0031 (14)	−0.0051 (13)
C9	0.0226 (14)	0.0217 (13)	0.0269 (15)	0.0030 (13)	0.0035 (12)	−0.0056 (12)
C10	0.0224 (15)	0.0189 (13)	0.0308 (16)	0.0038 (12)	0.0049 (12)	−0.0082 (12)
C11	0.0266 (15)	0.0265 (14)	0.0279 (15)	0.0035 (14)	0.0032 (13)	−0.0011 (12)
C12	0.0186 (13)	0.0196 (12)	0.0245 (14)	0.0049 (13)	0.0027 (11)	−0.0015 (11)
C13	0.0253 (14)	0.0225 (12)	0.0210 (13)	0.0052 (13)	0.0018 (12)	0.0020 (12)
C14	0.0261 (14)	0.0266 (13)	0.0222 (14)	0.0051 (13)	−0.0048 (13)	−0.0030 (12)
C15	0.0204 (14)	0.0199 (12)	0.0323 (15)	0.0044 (13)	0.0019 (12)	−0.0049 (12)
C16	0.0270 (15)	0.0247 (13)	0.0245 (14)	0.0028 (13)	0.0026 (12)	0.0029 (13)
C17	0.0297 (16)	0.0267 (13)	0.0204 (14)	0.0032 (14)	−0.0018 (12)	−0.0006 (12)
C18	0.0263 (15)	0.0271 (13)	0.0409 (17)	0.0013 (14)	−0.0003 (14)	−0.0062 (13)

### Geometric parameters (Å, °)

**Table:**

O1—C13	1.367 (3)	C7—H7	0.9500
O1—H1O	0.87 (3)	C8—C9	1.420 (3)
N1—C11	1.273 (3)	C8—H8	0.9500
N1—C12	1.414 (3)	C9—C10	1.427 (3)
C1—C2	1.377 (3)	C11—H11	0.9500
C1—C9	1.439 (3)	C12—C13	1.398 (3)
C1—C11	1.479 (3)	C12—C17	1.400 (3)
C2—C3	1.412 (3)	C13—C14	1.388 (3)
C2—H2	0.9500	C14—C15	1.387 (3)
C3—C4	1.365 (4)	C14—H14	0.9500
C3—H3	0.9500	C15—C16	1.401 (3)
C4—C10	1.411 (3)	C15—C18	1.507 (3)
C4—H4	0.9500	C16—C17	1.382 (4)
C5—C6	1.361 (3)	C16—H16	0.9500
C5—C10	1.424 (3)	C17—H17	0.9500
C5—H5	0.9500	C18—H18A	0.9800
C6—C7	1.402 (3)	C18—H18B	0.9800
C6—H6	0.9500	C18—H18C	0.9800
C7—C8	1.370 (3)		
			
C13—O1—H1O	109.9 (18)	C4—C10—C9	119.8 (2)
C11—N1—C12	121.1 (2)	C5—C10—C9	119.0 (2)
C2—C1—C9	119.2 (2)	N1—C11—C1	122.5 (2)
C2—C1—C11	120.1 (2)	N1—C11—H11	118.8
C9—C1—C11	120.7 (2)	C1—C11—H11	118.8
C1—C2—C3	121.3 (2)	C13—C12—C17	117.5 (2)
C1—C2—H2	119.3	C13—C12—N1	115.1 (2)
C3—C2—H2	119.3	C17—C12—N1	127.4 (2)
C4—C3—C2	120.5 (2)	O1—C13—C14	117.8 (2)
C4—C3—H3	119.8	O1—C13—C12	120.9 (2)
C2—C3—H3	119.8	C14—C13—C12	121.3 (2)
C3—C4—C10	120.5 (2)	C15—C14—C13	120.9 (2)
C3—C4—H4	119.7	C15—C14—H14	119.5
C10—C4—H4	119.7	C13—C14—H14	119.5
C6—C5—C10	121.3 (3)	C14—C15—C16	118.0 (2)
C6—C5—H5	119.4	C14—C15—C18	120.8 (2)
C10—C5—H5	119.4	C16—C15—C18	121.2 (2)
C5—C6—C7	119.8 (3)	C17—C16—C15	121.1 (2)
C5—C6—H6	120.1	C17—C16—H16	119.5
C7—C6—H6	120.1	C15—C16—H16	119.5
C8—C7—C6	120.9 (3)	C16—C17—C12	121.1 (2)
C8—C7—H7	119.6	C16—C17—H17	119.4
C6—C7—H7	119.6	C12—C17—H17	119.4
C7—C8—C9	121.1 (2)	C15—C18—H18A	109.5
C7—C8—H8	119.4	C15—C18—H18B	109.5
C9—C8—H8	119.4	H18A—C18—H18B	109.5
C8—C9—C10	117.9 (2)	C15—C18—H18C	109.5
C8—C9—C1	123.4 (2)	H18A—C18—H18C	109.5
C10—C9—C1	118.7 (2)	H18B—C18—H18C	109.5
C4—C10—C5	121.2 (2)		
			
C9—C1—C2—C3	0.3 (4)	C1—C9—C10—C5	179.3 (2)
C11—C1—C2—C3	179.4 (2)	C12—N1—C11—C1	−179.8 (2)
C1—C2—C3—C4	0.4 (4)	C2—C1—C11—N1	−0.5 (4)
C2—C3—C4—C10	−1.0 (4)	C9—C1—C11—N1	178.6 (2)
C10—C5—C6—C7	0.5 (4)	C11—N1—C12—C13	173.8 (2)
C5—C6—C7—C8	−1.3 (4)	C11—N1—C12—C17	−7.6 (4)
C6—C7—C8—C9	1.5 (4)	C17—C12—C13—O1	−179.6 (2)
C7—C8—C9—C10	−0.9 (3)	N1—C12—C13—O1	−0.9 (3)
C7—C8—C9—C1	180.0 (2)	C17—C12—C13—C14	1.2 (4)
C2—C1—C9—C8	178.7 (2)	N1—C12—C13—C14	179.9 (2)
C11—C1—C9—C8	−0.4 (4)	O1—C13—C14—C15	−178.7 (2)
C2—C1—C9—C10	−0.4 (3)	C12—C13—C14—C15	0.5 (4)
C11—C1—C9—C10	−179.5 (2)	C13—C14—C15—C16	−1.2 (3)
C3—C4—C10—C5	−178.6 (2)	C13—C14—C15—C18	178.8 (2)
C3—C4—C10—C9	0.9 (4)	C14—C15—C16—C17	0.2 (4)
C6—C5—C10—C4	179.5 (2)	C18—C15—C16—C17	−179.8 (2)
C6—C5—C10—C9	0.0 (4)	C15—C16—C17—C12	1.5 (4)
C8—C9—C10—C4	−179.4 (2)	C13—C12—C17—C16	−2.1 (4)
C1—C9—C10—C4	−0.2 (3)	N1—C12—C17—C16	179.3 (2)
C8—C9—C10—C5	0.1 (3)		

### Hydrogen-bond geometry (Å, °)

**Table:**

*D*—H···*A*	*D*—H	H···*A*	*D*···*A*	*D*—H···*A*
O1—H1O···O1^i^	0.87 (3)	2.17 (3)	2.916 (2)	143 (2)
O1—H1O···N1	0.87 (3)	2.24 (3)	2.701 (3)	113 (2)
C2—H2···O1^i^	0.95	2.53	3.382 (3)	150
C18—H18C···Cg^ii^	0.98	2.57	3.504 (3)	160

Symmetry codes: (i) *x*−1/2, −*y*+1/2, −*z*+1; (ii) *x*+1, *y*, *z*.
